# Pathotypes and probiotics: response to a commentary on the detection of a Shiga toxin producing *Escherichia coli* in a Crohn’s disease patient

**DOI:** 10.1186/s13099-015-0064-2

**Published:** 2015-07-11

**Authors:** Josias Rodrigues

**Affiliations:** Laboratory of Medical Bacteriology, Department of Microbiology and Immunology, Institute of Biosciences of the State University of São Paulo (UNESP), Distrito de Rubião Junior, Botucatu, SP CEP 18618-970 Brazil

## Abstract

A recent report on the detection in a Crohn’s disease (CD) patient of an adherent and invasive Shiga toxin producing *Escherichia coli* (STEC) (Gut pathogens 2015, 7:2) prompted a commentary expressing some skepticism on the significance of the paper findings (Gut pathogens 2015, 7:15). Besides focusing on recurrent issues concerning the difficulties in defining a pathogen, the opinion considers recent data demonstrating the presence of virulence factors in a commercial probiotic. In response to the commentary’s observations, additional information on the described STEC strain, as well as a short discussion on CD associated *E. coli* are presented here.

## Letter to the editor

The commentary by Wassenaar and Gunzer [1] on a recently published characterization study of a Crohn’s disease (CD) *Escherichia coli* combining virulence properties of multiple pathotypes [2] hits the complex issue on how to identify a pathogenic strain within this species, because of its high genetic and phenotypic heterogeneity and varying potential to cause disease. *E. coli* is a resident member of gut microbiota and a multi-disease organism alike, including a predominantly commensal and a smaller group of pathogenic strains [3]. The observation of a high numbers of *E. coli* in the gut of inflammatory bowel diseases (IBD) patients by many independent researchers in the last decades [4–6] has made these bacteria suspicious of involvement with these diseases as well. The main point expressed in the commentary is that the presence of virulence genes in an *E. coli* strain is not sufficient to grant it a role in disease because it has been found that pathogenic and probiotic strains of long recorded history of safe use share virulence genes and the administration of the later in a high dose to volunteers had no detrimental effects [7]. These findings are really important since they can instigate a discussion towards a better discernment of what among the many known *E. coli* traits can be considered as true virulence factors. Yet, in my view the data are still modest to sustain any conclusion and care must be taken to avoid generalizations. The number of virulence genes detected in the probiotics was limited and in general restricted to factors involved in colonization and fitness (toxins genes, for example, were not found). The comparison of EDL933 strain’s proteome with that of G3/10, one of the probiotic strain used in the study, showed that the protein genes typical of the former were mostly absent in the probiotic *E. coli* [7]. Analysis of genetic relationship put probiotics and commensal or non-pathogenic strains in a single cluster apart from *E. coli* reference pathogenic strains.

The commentary on da Silva Santos et al.’s paper seems not to consider that the focus of the original work was not restricted to investigate *genetic traits*, but also virulence associated *phenotypes*, notably the capacity to adhere and to invade epithelial cells. *E. coli* D92/09 strain was able to replicate inside macrophages and its efficiency of entry into these cells was higher than that shown by adherent and invasive *E. coli* (AIEC) strain LF82 [2]. Innumerous studies published to date [5, 8, 9] have demonstrated a high prevalence of adherent strains among *E. coli* from CD patients. The characterization of the invasive phenotype of a strain from one of these works [10] and subsequent studies [11] led to the description of the AIEC pathotype. Evidence gathered from these studies settled the basis for the recognition of AIEC as the most probable bacterial agent suspected of playing an active role in CD [12]. Concerning the strain described by da Silva Santos et al. [2], in addition to the adherent and invasive phenotype, the bacteria proved to be lethal to the host cell. This and other features reveal a virulence profile distinct from those expressed by AIEC (Table 1) and whose significance should be investigated in experiments with animal models. In addition, search for strains bearing markers similar to those observed in *E. coli* D92/09 in other CD patients should be done. In a recent PCR screening [13, 14] performed in my lab to detect some *E. coli* O serogroups in a bacterial collection from nine CD patients and eight control subjects, who routinely attended the São Paulo State University Hospital (HC-UNESP), a high prevalence of O25, O83 and O126 could be observed in both groups. One of these O25 strains, detected in a CD patient, belonged to the O25:H4 serotype and ST131, an ESBL producing virulent clone of global distribution [15, 16]. Strains of this clone bearing AIEC phenotype have been identified among both intestinal and extraintestinal pathogenic *E. coli* (ExPEC) [17]. O25 and O83, but not O126, include multidrug resistant serotypes frequently associated with nosocomial infections [18]. D92/09 strain belongs to O126:H27 (Table 1), an O:H type traditionally involved with diarrheal diseases [19], but which in this case marked a CD clinical isolate. Manual search in this strain’s genome revealed genes conferring resistance to aminoglycoside (*strB*), beta-lactam (*bla*_TEM-1B_), fluoroquinolone (*Qnr*B19), tetracycline (*tet*(A)) and trimethoprim (*dfr*A8). This finding allied to the high prevalence of O126 strains among the patients, as mentioned above, could indicate that possibly strains within this serogroup can be involved with nosocomial infections in HC-UNESP and that D92/09 strain may have infected the patient in the hospital environment. Since many CD *E. coli* strains belong to serotypes of ExPEC [17], some of which associated with nosocomial infections, it is likely that by attending the hospital in a routine basis, it is reasonable to suppose that at least some of the CD patients may acquire infections by these bacteria in the hospital settings.

**Table 1:** Some features of *E. coli* D92/09 strain in comparison with AIEC LF82

Feature	D92/09	LF82
Origin	Ileal biopsy and stool from an Ileal resected adult female CD patient [2]	Ileal biopsy from an Ileal resected adult female CD patient [5]
Cell interaction	Aggregative adhesion to cultured epithelial cells; invasion in Hep-2 and Caco-2 cells^a^; high invasive ability with discrete replication in macrophage J774; invasiveness associated with cytotoxic effect [2]	Diffuse adhesion to cultured epithelial cells [5]; invasion in several cell types, such as HEp-2 [10], Caco-2 and Int-407 [5]; invasion and replication in macrophage J774 [21]; do not kill host cells [10]
Typing	Serotype O126:H27; EcoR phylogroup B2; ST 3057 [2]	Serotype O83:H1; EcoR phylogroup B2 [22]; ST135 [17]
Genome size	4.94 Mbp [2]	4.88 Mbp [23]
Pathotype	STEC	AIEC

^a^No other epithelial cell type was tested.

A significant consideration in the commentary refers to the absence of symptoms in the infected patient, a condition which was explained by the existence of an immune system control, preventing bacterial action [7]. Indeed, this might have been the case but not necessarily is to mean that the strain is devoid of any virulence potential. It has been shown that the abundance of *E. coli* in the ileal mucosa of post-operative CD patients may not be linked to disease activity [4, 5]. Nonetheless, the D92/09 host presented discrete clinical activity at the site of bacterial isolation insufficient to produce detectable symptoms [2].

Concerning the involvement in CD, doubts remain not only in regard to D92/09 strain but also to *E. coli* as a whole. Many of the disease provoked by this species are attributable to opportunists sharing colonization factors of pathogenic strains. Abnormal proliferation and involvement with disease could depend on additional and particular bacterial properties which could act in specific predisposing condition of the host. That seems to be the case of CD patients whose ileal enterocytes overexpress carcinoembryonic antigen-related cell adhesion molecule 6 (CEACAM6) which was shown to be a receptor for AIEC type 1 pili [20]. In theory, this predisposing condition would also favor strains of the probiotics such as Symbioflor2, possessing type 1 pili. A number of key factors described in LF82 strain, not present in the probiotics, such as the ability to replicate inside macrophages and potential to form granulomas constitute evidence strong enough to suspect of the participation of AIEC in a disease with the clinical and histopathological features typical of CD. In other words, the manifestation of virulence depends on the concerted action of multiple factors. Although not fully characterized, the available information on D92/09 strain, configures a profile with potential do cause damage in a condition of opportunism such as that in CD [2].

In conclusion, both the Wassenaar and Gunzer’s commentary on D92/09 strain and the results of their recently published work on virulence genes in probiotics are very interesting and should represent a starting point on a discussion which may broaden our knowledge on the different ways that *E. coli* interacts with humans, as probiotics, commensals or pathogens.

## References

[1] Wassenaar TM, Gunzer F (2015). The prediction of virulence based on presence of virulence genes in *E. coli* may not always be accurate. Gut Pathog.

[2] da Silva Santos AC, Gomes Romeiro F, Yukie Sassaki L, Rodrigues J (2015). *Escherichia coli* from Crohn’s disease patient displays virulence features of enteroinvasive (EIEC), enterohemorragic (EHEC), and enteroaggregative (EAEC) pathotypes. Gut pathog.

[3] Kaper JB, Nataro JP, Mobley HL (2004). Pathogenic *Escherichia coli*. Nat Rev Microbiol.

[4] Keighley MR, Arabi Y, Dimock F, Burdon DW, Allan RN, Alexander-Williams J (1978). Influence of inflammatory bowel disease on intestinal microflora. Gut.

[5] Darfeuille-Michaud A, Neut C, Barnich N, Lederman E, Di Martino P, Desreumaux P (1998). Presence of adherent *Escherichia coli* strains in ileal mucosa of patients with Crohn’s disease. Gastroenterology.

[6] de Souza HL, de Carvalho VR, Romeiro FG, Sassaki LY, Keller R, Rodrigues J (2012). Mucosa-associated but not luminal *Escherichia coli* is augmented in Crohn’s disease and ulcerative colitis. Gut pathog.

[7] Wassenaar TM, Zschuttig A, Beimfohr C, Geske T, Auerbach C, Cook H (2015). Virulence genes in a probiotic *E. coli* product with a recorded long history of safe use. Eur J Microbiol Immunol.

[8] Burke DA, Axon AT (1988). Adhesive *Escherichia coli* in inflammatory bowel disease and infective diarrhoea. BMJ.

[9] Thomazini CM, Samegima DA, Rodrigues MA, Victoria CR, Rodrigues J (2011). High prevalence of aggregative adherent *Escherichia coli* strains in the mucosa-associated microbiota of patients with inflammatory bowel diseases. IJMM.

[10] Boudeau J, Glasser AL, Masseret E, Joly B, Darfeuille-Michaud A (1999). Invasive ability of an *Escherichia coli* strain isolated from the ileal mucosa of a patient with Crohn’s disease. Infect Immun.

[11] Rolhion N, Darfeuille-Michaud A (2007). Adherent-invasive *Escherichia coli* in inflammatory bowel disease. Inflamm Bowel Dis.

[12] Agus A, Massier S, Darfeuille-Michaud A, Billard E, Barnich N (2014). Understanding host-adherent-invasive interaction in Crohn’s disease: opening up new therapeutic strategies. BioMed Res Intl.

[13] Li D, Liu B, Chen M, Guo D, Guo X, Liu F (2010). A multiplex PCR method to detect 14 *Escherichia coli* serogroups associated with urinary tract infections. J Microbiol Methods.

[14] Liu Y, DebRoy C, Fratamico P (2007). Sequencing and analysis of the *Escherichia coli* serogroup O117, O126, and O146 O-antigen gene clusters and development of PCR assays targeting serogroup O117-, O126-, and O146-specific DNA sequences. Mol Cell Probes.

[15] Johnson JR, Urban C, Weissman SJ, Jorgensen JH, Lewis JS, Hansen G (2012). Molecular epidemiological analysis of *Escherichia coli* sequence type ST131 (O25:H4) and blaCTX-M-15 among extended-spectrum-beta-lactamase-producing *E. coli* from the United States, 2000–2009. Antim Agents Chemoth.

[16] Blanco M, Alonso MP, Nicolas-Chanoine MH, Dahbi G, Mora A, Blanco JE (2009). Molecular epidemiology of *Escherichia coli* producing extended-spectrum {beta}-lactamases in Lugo (Spain): dissemination of clone O25b:H4-ST131 producing CTX-M-15. J Antimicrob Chem.

[17] Martinez-Medina M, Mora A, Blanco M, Lopez C, Alonso MP, Bonacorsi S (2009). Similarity and divergence among adherent-invasive *Escherichia coli* and extraintestinal pathogenic *E. coli* strains. J Clin Microbiol.

[18] Momtaz H, Karimian A, Madani M, Safarpoor Dehkordi F, Ranjbar R, Sarshar M (2013). Uropathogenic *Escherichia coli* in Iran: serogroup distributions, virulence factors and antimicrobial resistance properties. Ann Clin Microbiol Antim.

[19] Yam WC, Robins-Browne RM, Lung ML (1994). Genetic relationships and virulence factors among classical enteropathogenic *Escherichia coli* serogroup O126 strains. J Med Microbiol.

[20] Barnich N, Carvalho FA, Glasser AL, Darcha C, Jantscheff P, Allez M (2007). CEACAM6 acts as a receptor for adherent-invasive *E. coli*, supporting ileal mucosa colonization in Crohn disease. J Clin Invest.

[21] Glasser AL, Boudeau J, Barnich N, Perruchot MH, Colombel JF, Darfeuille-Michaud A (2001). Adherent Invasive *Escherichia coli* strains from patients with Crohn’s disease survive and replicate within macrophages without inducing host cell death. Infect Immun.

[22] Darfeuille-Michaud A, Boudeau J, Bulois P, Neut C, Glasser AL, Barnich N (2004). High prevalence of adherent-invasive *Escherichia coli* associated with ileal mucosa in Crohn’s disease. Gastroenterology.

[23] Miquel S, Peyretaillade E, Claret L, de Vallee A, Dossat C, Vacherie B (2010). Complete genome sequence of Crohn’s disease-associated adherent-invasive *E. coli* strain LF82. PloS One.

